# Supplementary material for: RNA targeting and cleavage by the type III-Dv CRISPR effector complex
Source: Nat Commun. 2024 Apr 18;15:3324. doi: 10.1038/s41467-024-47506-y (PMC11026444; doi:10.1038/s41467-024-47506-y)
Supplement: Supplementary file 1 — Supplementary Information [file 41467_2024_47506_MOESM1_ESM.pdf]

Supplementary Information

RNA targeting and cleavage by the type III-Dv CRISPR effector complex

Evan A. Schwartz, Jack P.K. Bravo, Mohd Ahsan, Luis A. Macias, Caitlyn L. McCafferty, Tyler L. Dangerfield, Jada N. Walker, Jennifer S. Brodbelt, Giulia Palermo, Peter C. Fineran, Robert D. Fagerlund, and David W. Taylor

Supplementary Table 1 | Theoretical masses, experimental masses, and % deviation of the type III-Dv complex, subcomplexes, and individual subunits. Experimental masses were recorded via native mass spectrometry and denaturing LC-mass spectrometry.

Type III-Dv Complex	Theoretical mass (Da)	Native MS Experimental mass (Da)	Denaturing LC-MS Experimental mass (Da)	Deviation from expected mass (%)*
Cas7_insert•Cas7-Cas5-Cas11•Cas10-His6•Cas7-2x•Csx19•mature crRNA	331,405	332,300	-	0.27
Cas7_insert•Cas7-Cas5-Cas11•Cas10-His6•Cas7-2x•mature crRNA	310,213	310,980	-	0.25
Cas7_insert•Cas7-Cas5-Cas11•Cas10-His6•Csx19•mature crRNA	274,778	274,840	-	0.02
Cas7_insert•Cas7-Cas5-Cas11•Cas7-2x•Csx19•mature crRNA	267,286	267,770	-	0.18
Cas10-His6•mature crRNA	76,055	-	76,110	0.07
Cas7_insert	90,080	-	90,080	0.00
Cas7-Cas5-Cas11	87,451	-	87,450	0.00
Cas10-His6	64,119	64,170	64,120	0.00
Cas7-2x	56,627	-	56,630	0.01
Csx19	21,192	21,190	21,190	0.01
Mature crRNA	11,936	-	-	-

Supplementary Table 2 | Model statistics for the type III-Dv binary complex, non-self target-bound complex, self target pre-cleavage complex, and post-cleavage complex.

	Binary Complex	Non-Self Target-Bound Complex	Self Target Pre-Cleavage Complex	Self Target Post-cleavage complex
**Data collection and processing**	cryoSPARC v2	cryoSPARC v2	cryoSPARC v3	cryoSPARC v3
Magnification	105,000x	105,000x	105,000x	105,000x
Voltage (kV)	300	300	300	300
Electron exposure (e–/Å^2)	80.5	80.5	80	80
Defocus range (μm)	1.2-2.2	1.2-2.2	1.2-2.2	1.2-2.2
Pixel size (Å)	0.81	0.81	0.8332	0.8332
Symmetry imposed	C1	C1	C1	C1
Initial particle images (no.)	1,890,840	1,919,796	1,280,364	1,280,364
Final particle images (no.)	648,782	609,722	181,656	41,202
Map resolution (Å)<br>FSC threshold	2.5<br>0.143	2.8<br>0.143	3.0<br>0.143	3.4<br>0.143
Map resolution range (Å)	N/A	N/A	N/A	N/A
**Refinement**				
Model resolution (Å)<br>FSC threshold	2.6<br>0.5	2.8<br>0.5	3.1<br>0.5	3.7<br>0.5
Model resolution range (Å)	N/A	N/A	N/A	N/A
Map sharpening *B* factor (Å^2)	99.3	124.7	114.1	97.5
Model composition<br>Non-hydrogen atoms<br>Protein residues<br>Ligands	 21968<br>2692<br>0	 22598<br>2685<br>0	 22589<br>2685<br>0	 22405<br>2697<br>0
B factors (Å^2)<br>Protein<br>Nucleotide	 14.56/101.45/47.39<br>15.79/67.58/30.46	 15.07/97.79/50.92<br>16.14/80.16/36.66	 13.69/81.92/40.10<br>12.49/79.61/28.83	 46.76/162.27/88.37<br>54.04/126.03/72.68
R.m.s. deviations<br>Bond lengths (Å)<br>Bond angles (°)	 0.008<br>0.894	 0.007<br>0.866	 0.005<br>0.786	 0.006<br>0.801
**Validation**<br>MolProbity score<br>Clashscore<br>Poor rotamers (%)	 1.65<br>4.54<br>1.85	 1.66<br>5.19<br>1.37	 1.50<br>2.10<br>2.08	 1.59<br>3.15<br>1.76
Ramachandran plot<br>Favored (%)<br>Allowed (%)<br>Disallowed (%)	 96.60<br>3.18<br>0.22	 95.91<br>3.79<br>0.30	 96.02<br>3.71<br>0.26	 95.71<br>3.88<br>0.41

Supplementary Table 3: Plasmids used in this study.

Plasmid	Description	Reference
pACYCDuet-1	Two T7/LacO promoters with P15A replicon, Cm^R	Novagen
pPF2434	N-His_6-tagged Cas10, Cas7-5-11, Cas7_2x, Csx19 and Cas7-insert, pRSF-1b	This study
pPF2441	Spacer1 of Synechocystis type III-Dv CRISPR array, pACYCDuet-1	This study
pPF2442	Plasmid pPF2441 with Cas6-2a	This study
pPF3085	Modified pPF2434 with Cas7_2x(D29A,D31A, D33A)	This study
pPF3086	Modified pPF2434 with Cas7_2x(D241A,D246A)	This study
pPF3089	Modified pPF2434 without Csx19	This study
pPF3205	Modified pPF2434 with Cas7-5-11(D26A)	This study
pPF3436	Modified pPF2434 with Cas10(H487A)	This study
pPF3519	Modified pPF2434 with Cas10(R490A)	This study
pPF3521	Modified pPF2434 with Cas7-5-11(D678A)	This study
pPF3522	Modified pPF2434 with Cas7-5-11(D769A)	This study
pPF3636	Modified pPF2434 with Cas10(H487A,R490A)	This study
pRSF-1b	T7/LacO promoter with RSF1030-derived replicon, Km^R	Novagen

Supplementary Table 4: Oligonucleotides used in this study.

DNA oligonucleotides (restriction sites are underlined)

Name	Sequence (5'-3')	Notes
PF4847	TATACATATGGCATACAGACTGTTTTTCAGTGTGATAG	F repeats and spacer1
PF4848	CGAGGGTACCGGGACTCCAACCCCCCAAG	R repeats and spacer1
PF4849	TATACCATGGTGGATCTAAAATCCTTAGCTG	F *cas6-2a*
PF4850	ATTCGGATCCTTATTGAACATTGGCTAAGGC	R *cas6-2a*
PF4851	TGGGTACCGAAAACCTGTATTTTCAGGGCTTTCTAGTTCTAATTGAGACTTCCGGTAATC	F *cas10*
PF4852	CGGCCGCAAGCTTGTCGACCTGCAGTTAACTAGGTTTGATTGGAAAACTCTGG	R *cas7-insert*
PF5991	GGCGCCGCTGCGACGGCTTTAGCCCTGGCGGTTAATGGTG	F *cas7-2x* D33A mutant
PF5992	AAAGCCGTCGCAGCGGCGCCACCCACACCACCAATG	R *cas7-2x* D33A mutant
PF5993	GGCTGGACTGGCGATCGCTATTTTGCCCCTCGTTAGTCAAGTG	F *cas7-2x* D246A mutant
PF5994	TAGCGATCGCCAGTCCAGCCCCTTCAGCTTTCACCATGAC	R *cas7-2x* D246A mutant
PF6281	GCCTAAGTTAGTAACTTTACCACTACCACCAATATGAAATTACCCTC	F Δ*csx19* mutant
PF6282	GTAAAGTTACTAACTTAGGCGGCCTCCTGCTG	R Δ *csx19* mutant
PF6423	GAACTAGCCAGTGTTGTACAACGGGATGGAG	F *cas7-5-11* D26A mutant
PF6424	TGTACAACACTGGCTAGTTCCCCCCGACCCATG	R *cas7-5-11* D26A mutant
PF6983	AGTCAAAGCGCTGCCCTACGAACGGCATTG	F *cas10* H487A mutant
PF6984	CGTAGGGCAGCGCTTTGACTGCTGGGTAATGC	R *cas10* H487A mutant
PF6994	GCCCTAGCAACGGCATTGTACCTAGAGAAAAATG	F *cas10* R490A mutant
PF6995	TACAATGCCGTTGCTAGGGCATGGCTTTGACTG	R *cas10* R490A mutant
PF6998	GGAGGATTTGCCTCCGTATTAAAACGGCTACACTC	F *cas7-5-11* D678A mutant
PF6999	AATACGGAGGCAAATCCTCCTAATTGAGTCATGGTG	R *cas7-5-11* D678A mutant

Name	Sequence (5'-3')	Notes
PF7000	CTATCATTGCGGGTCATAAACGGGACTTAGAAAAAG	F *cas7-5-11* D769A mutant
PF7001	TTTATGACCCGCAATGATAGCGTCAACAAGGGTTC	R *cas7-5-11* D769A mutant
PF7305	TGCCCTAGCAACGGCATTGTACCTAGAGAAAAATG	F *cas10* H487A,R490A mutant
PF7306	ACAATGCCGTTGCTAGGGCAGCGCTTTGACTG	R *cas10* H487A,R490A mutant

RNA oligonucleotides

PF5855	rCrArUrGrArCrGrGrArUrCrGrCrGrGrGrArGrUrUrArUrUrGrArCrGrArCrCrCrCrGrArUrUrGrGrUrUrCrUrArCrUrArCrArArArCrGrUrGrArUrArCrUrA	60nt RNA target
PF5856	/5IRD800CWN/rCrArUrGrArCrGrGrArUrCrGrCrGrGrGrArGrUrUrArUrUrGrArCrGrArCrCrCrCrGrArUrUrGrGrUrUrCrUrArCrUrArCrArArArCrGrUrGrArUrArCrUrA	5'-IRD800 60nt RNA target
PF6575	/56-FAM/rCrArUrGrArCrGrGrArUrCrGrCrGrGrGrArGrUrUrArUrUrGrArCrGrArCrCrCrCrGrArUrUrGrGrUrUrCrUrArCrUrArCrArArArCrGrUrGrArUrArCrUrA	5'-FAM 60nt RNA target
PF6576	rCrArUrGrArCrGrGrArUrCrGrCrGrGrGrArGrUrUrArUrUrGrArCrGrArCrCrCrCrGrArUrUrGrGrUrUrCrUrArCrUrArCrArArArCrGrUrGrArUrArCrUrA/36-FAM/	3'-FAM 60nt RNA target
PF6577	/56-FAM/rCrArUrGrArCrGrGrArUrCrGrCrGrGrGrArGrUrUrArUrUrGrArCrGrArCrCrCrCrGrArUrUrGrGrUrUrCrUrA	5'-FAM 43nt RNA target
PF6578	/56-FAM/rCrArUrGrArCrGrGrArUrCrGrCrGrGrGrArGrUrUrArUrUrGrArCrGrArCrCrCrCrGrArUrUrG	5'-FAM 37nt RNA target
PF6579	/56-FAM/rCrArUrGrArCrGrGrArUrCrGrCrGrGrGrArGrUrUrArUrUrGrArCrGrArCrCrC	5'-FAM 31nt RNA target
PF6580	/56-FAM/rCrArUrGrArCrGrGrArUrCrGrCrGrGrGrArGrUrUrArUrUrGrArCrG	5'-FAM 27nt RNA target
PF6582	/56-FAM/rCrArUrGrArCrGrGrArUrCrGrCrGrGrGrArGrUrUrArUrUrGrArCrGrArCrCrCrCrGrArUrUrGrGrUrUrCrUrArCrUrArCrArGrUrUrUrCrArGrUrCrCrCrC	5'-FAM 60nt RNA anti-repeat
PF6583	/5IRD800CWN/rCrArUrGrArCrGrGrArUrCrGrCrGrGrGrArGrUrUrArUrUrGrArCrGrArCrCrCrCrGrArUrUrG	5'-IRD800 37nt RNA target
PF6971	/56-FAM/rCrArUrGrArCrGrGrArUrCrGrCrGrGrGrArGrUrArUrArArCrUrCrGrArCrCrCrCrGrArUrUrGrGrUrUrCrUrArCrUrArCrArArArCrGrUrGrArUrArCrUrA	5'-FAM 60nt RNA target, mismatch 6nt seed

Name	Sequence (5'-3')	Notes
PF6972	/56-FAM/rCrArUrGrArCrGrGrArUrCrGrCrGrGrGrArGrUrUrArUrUrGrArCrGrArCrCrCrCrGrArUrUrGrGrUrUrCrUrUrGrArUrGrUrArArCrGrUrGrArUrArCrUrA	5'-FAM 60nt RNA target, mismatch 6nt distal
PF6973	/56-FAM/rCrArUrGrArCrGrGrArUrCrGrCrGrGrGrArGrUrArUrArUrGrArCrGrArCrCrCrCrGrArUrUrGrGrUrUrCrUrArCrUrArCrArArArCrGrUrGrArUrArCrUrA	5'-FAM 60nt RNA target, mismatch 3nt 5' seed
PF6974	/56-FAM/rCrArUrGrArCrGrGrArUrCrGrCrGrGrGrArGrUrUrArUrArCrUrCrGrArCrCrCrCrGrArUrUrGrGrUrUrCrUrArCrUrArCrArArArCrGrUrGrArUrArCrUrA	5'-FAM 60nt RNA target, mismatch 3nt 3' seed
PF6975	/56-FAM/rCrArUrGrArCrGrGrArUrCrGrCrGrGrGrArGrUrUrArUrUrGrArCrGrArCrCrCrCrGrArUrUrGrGrUrUrCrUrArCrUrUrGrUrArArCrGrUrGrArUrArCrUrA	5'-FAM 60nt RNA target, mismatch 3nt 3' distal
PF7419	/5IRD800CWN/rCrArUrGrArCrGrGrArUrCrGrCrGrGrGrArGrUrUrArUrUrGrArCrGrArCrCCrCrGrArUrUGrGrUrUrCrUArCrUrArCrArArArCrGrUrGrArUrArCrUrA	5'-IRD800 60nt RNA target, A43+G37+C31 without 2'-OH
PF7420	/5IRD800CWN/rCrArUrGrArCrGrGrArUrCrGrCrGrGrGrArGrUrUrArUrUrGrArCrGrArCrCCrCrGrArUrUrGrGrUrUrCrUrArCrUrArCrArArArCrGrUrGrArUrArCrUrA	5'-IRD800 60nt RNA target, C31 without 2'-OH
PF7421	/5IRD800CWN/rCrArUrGrArCrGrGrArUrCrGrCrGrGrGrArGrUrUrArUrUrGrArCrGrArCrCrCrCrGrArUrUGrGrUrUrCrUrArCrUrArCrArArArCrGrUrGrArUrArCrUrA	5'-IRD800 60nt RNA target, G37 without 2'-OH
PF7422	/5IRD800CWN/rCrArUrGrArCrGrGrArUrCrGrCrGrGrGrArGrUrUrArUrUrGrArCrGrArCrCrCrCrGrArUrUrGrGrUrUrCrUArCrUrArCrArArArCrGrUrGrArUrArCrUrA	5'-IRD800 60nt RNA target, A43 without 2'-OH

Supplementary Table 5: Bacterial strains used in this study.

Strain	Genotype/Phenotype/Description	Reference
DH5α	*E. coli* F^-, ϕ80d*lacZ*ΔM15, Δ(*lacZYA–argF*)U169, *endA1*, *recA1*, *hsd*R17 ($r_K^- m_K^+$), *deoR*, *thi-1*, *sup*E44, λ^-, *gyr*A96, *rel*A1	Gibco/BRL
LOBSTR	*E. coli* B F^- *ompT, gal, dcm, lon, hsdS*$_B$($r_B^- m_B^-$), λ(DE3 [*lacI lacUV5-T7p07 ind1 sam7 nin5*]) [*malB*$^+$]$_{K-12}$(λ^S) *arnA slyD*	Kerafast
Synechocystis sp. PCC 6803	Glucose tolerant laboratory wild-type strain GT-01	(Morris et al., 2014), (Williams, 1988)

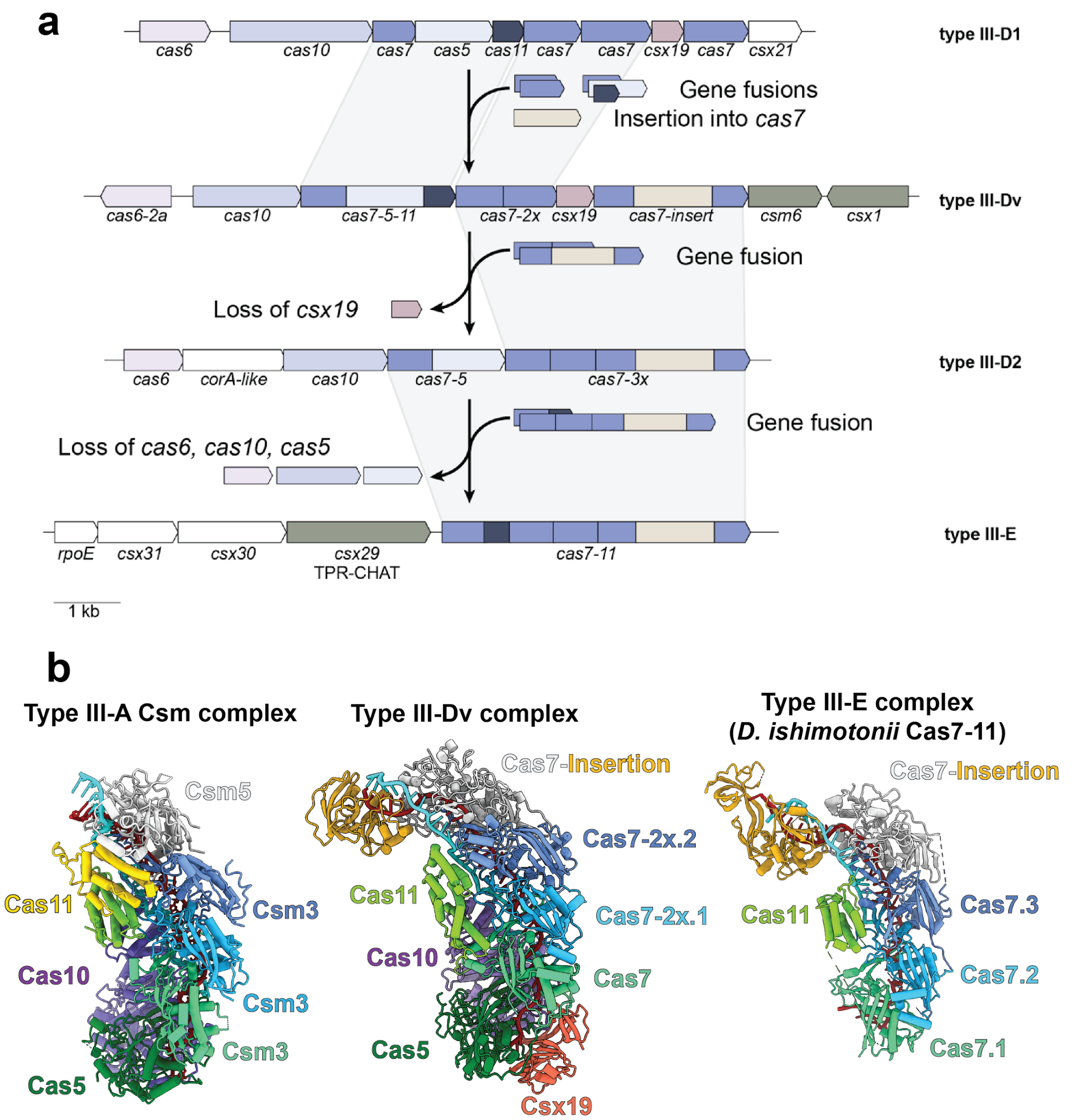

Supplementary Fig. 1 | Proposed evolution of type III CRISPR-Cas systems. a, Proposed evolutionary progression from type III-D1 to type III-E, with other multi-subunit type III systems presumably preceding type III-D1. **b,** Structural comparison between the multi-subunit type III-A (Csm) complex (PDB: 6IFY), the multi-subunit type III-Dv complex containing many subunit fusions, and the single-subunit type III-E complex (PDB: 7WAH).

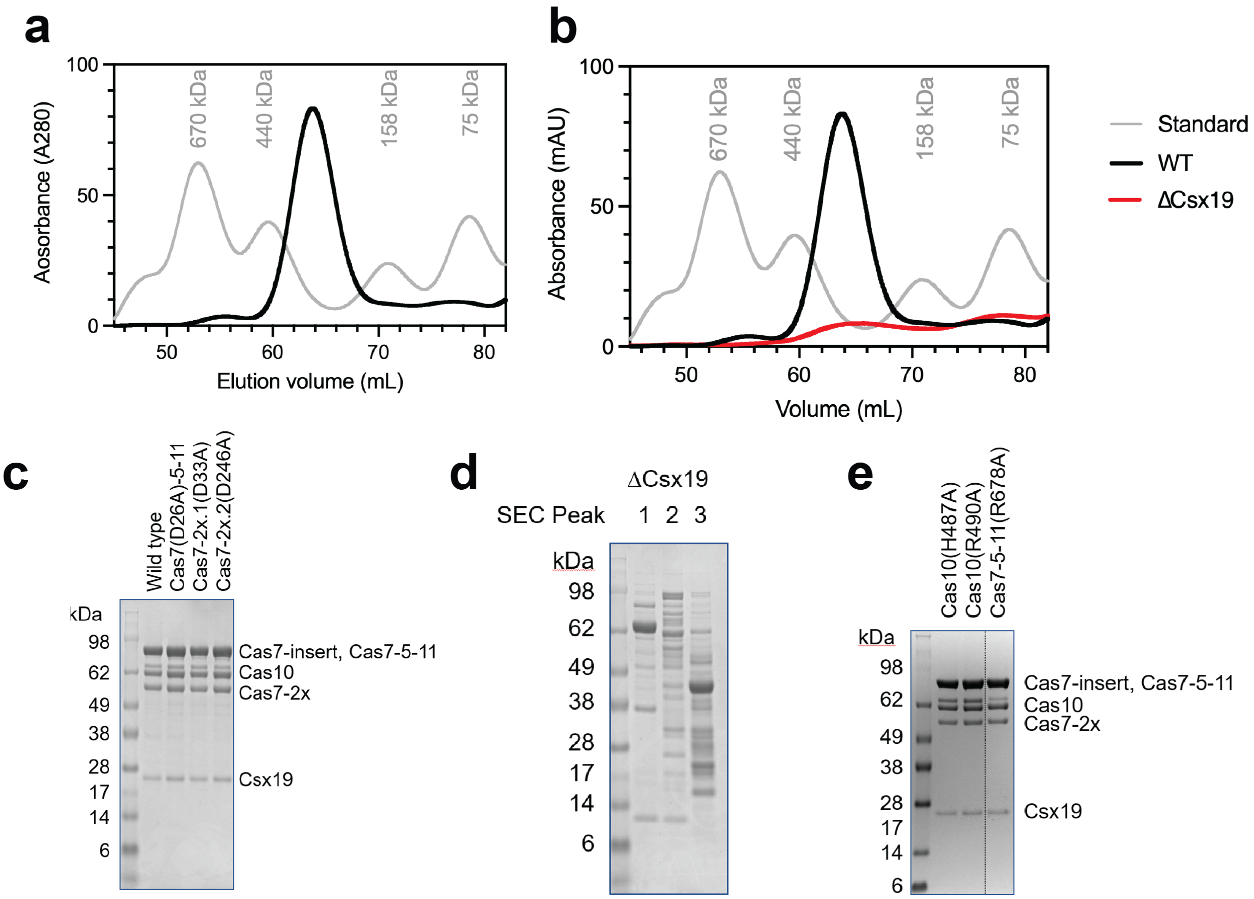

Supplementary Fig. 2 | Purification of the type III-Dv effector complex. a, Size-exclusion chromatograph of the WT type III-Dv complex. Black peak corresponds to ~330 kDa. Grey peaks represent standardized molecular weights. **b,** Size-exclusion chromatograph of the ΔCsx19 type III-Dv complex purification (red trace). Black peak corresponds to wild-type III-Dv complex. Grey peaks represent standardized molecular weights. **c,** Purification of Cas7 active site aspartate mutants for RNA cleavage analysis. **d,** SDS-PAGE of the ΔCsx19 from the two broad peaks seen in **b**. No complex appeared to form. **e,** Purification of other Cas7 active site residues predicted to be involved in RNA cleavage acid-base catalysis.

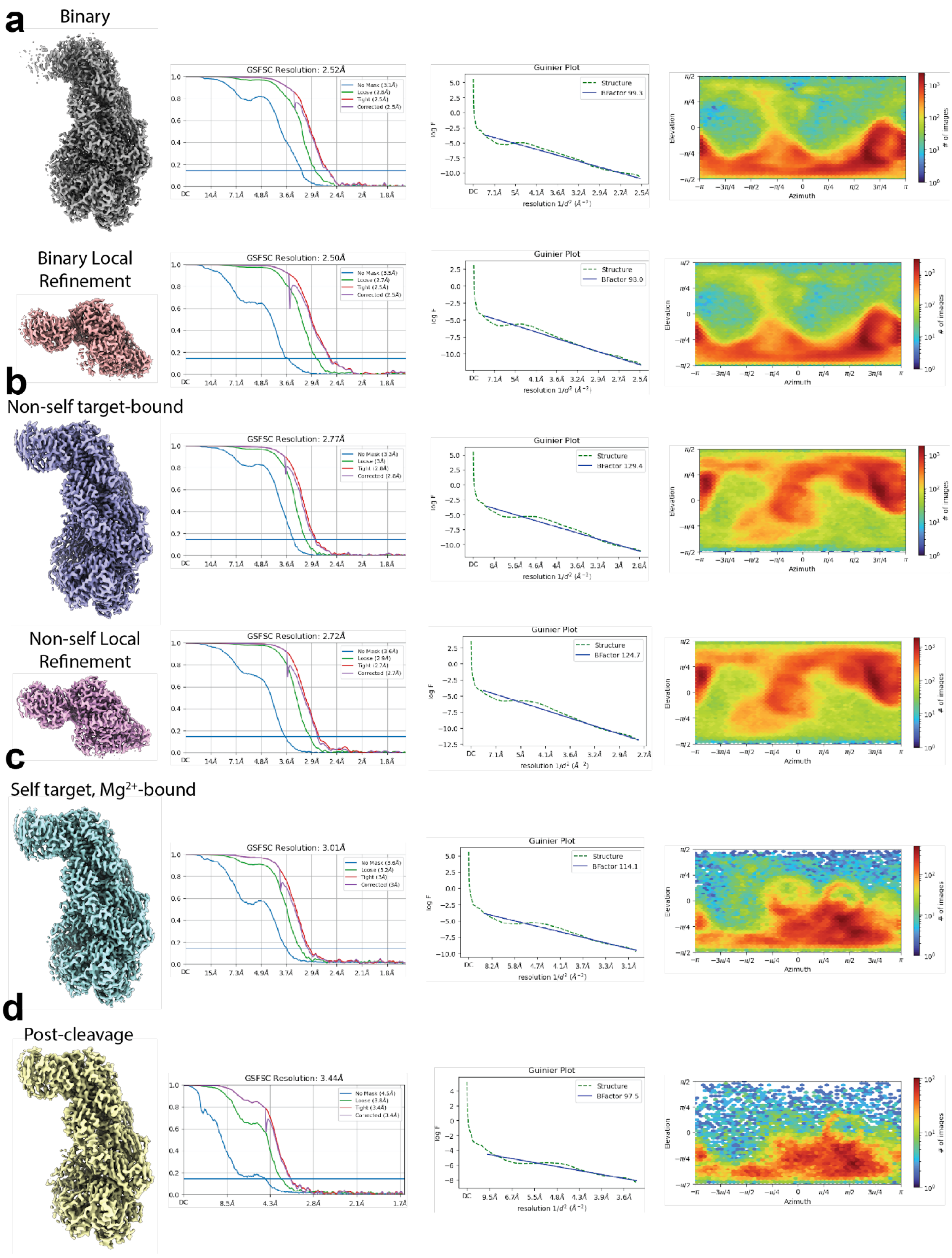
a
Binary
GSFSC Resolution: 2.52Å
Guinier Plot
Binary Local Refinement
GSFSC Resolution: 2.50Å
Guinier Plot
b
Non-self target-bound
GSFSC Resolution: 2.77Å
Guinier Plot
Non-self Local Refinement
GSFSC Resolution: 2.72Å
Guinier Plot
c
Self target, Mg2+-bound
GSFSC Resolution: 3.01Å
Guinier Plot
d
Post-cleavage
GSFSC Resolution: 3.44Å
Guinier Plot

Supplementary Fig. 3 | EM validation of type III-Dv maps. Final reconstructions, FSC plots for resolution determination, Guinier plot for b-factor sharpening, and directional distribution plots of the type III-Dv **a,** binary consensus (top) and local refinement (bottom) structures **b,** non-self target-bound consensus and local refinement structures **c,** Self target and Mg^{2+}-bound structure and **d,** Self target-bound post-cleavage structure.

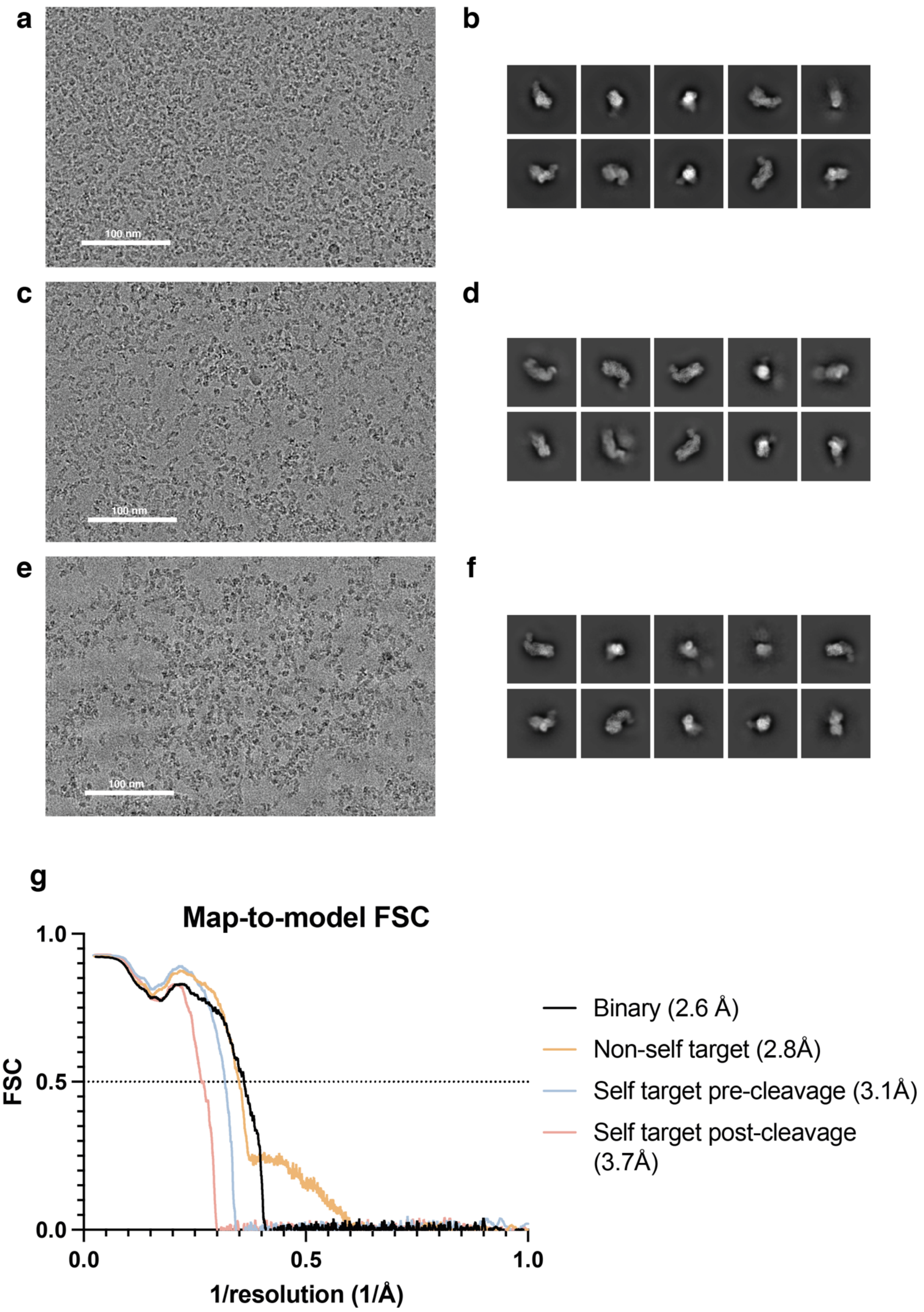
a
b
c
d
e
f
g
100 nm
100 nm
100 nm
Map-to-model FSC
FSC
1.0
0.5
0.0
0.0
0.5
1.0
1/resolution (1/Å)
Binary (2.6 Å)
Non-self target (2.8Å)
Self target pre-cleavage (3.1Å)
Self target post-cleavage (3.7Å)

Supplementary Fig. 4 | Additional cryo-EM analysis of III-Dv complexes. a & b, Cryo-EM micrograph and 2D class averages of binary complex. **c & d,** Cryo-EM micrograph and 2D class averages of non-self target-bound. **e & f,** Cryo-EM micrograph and 2D class averages of self-target + Mg^{2+}. **g:** Map-to-model FSC curves of binary, non-self target Mg^{2+}-bound, and post-cleavage complexes, respectively.

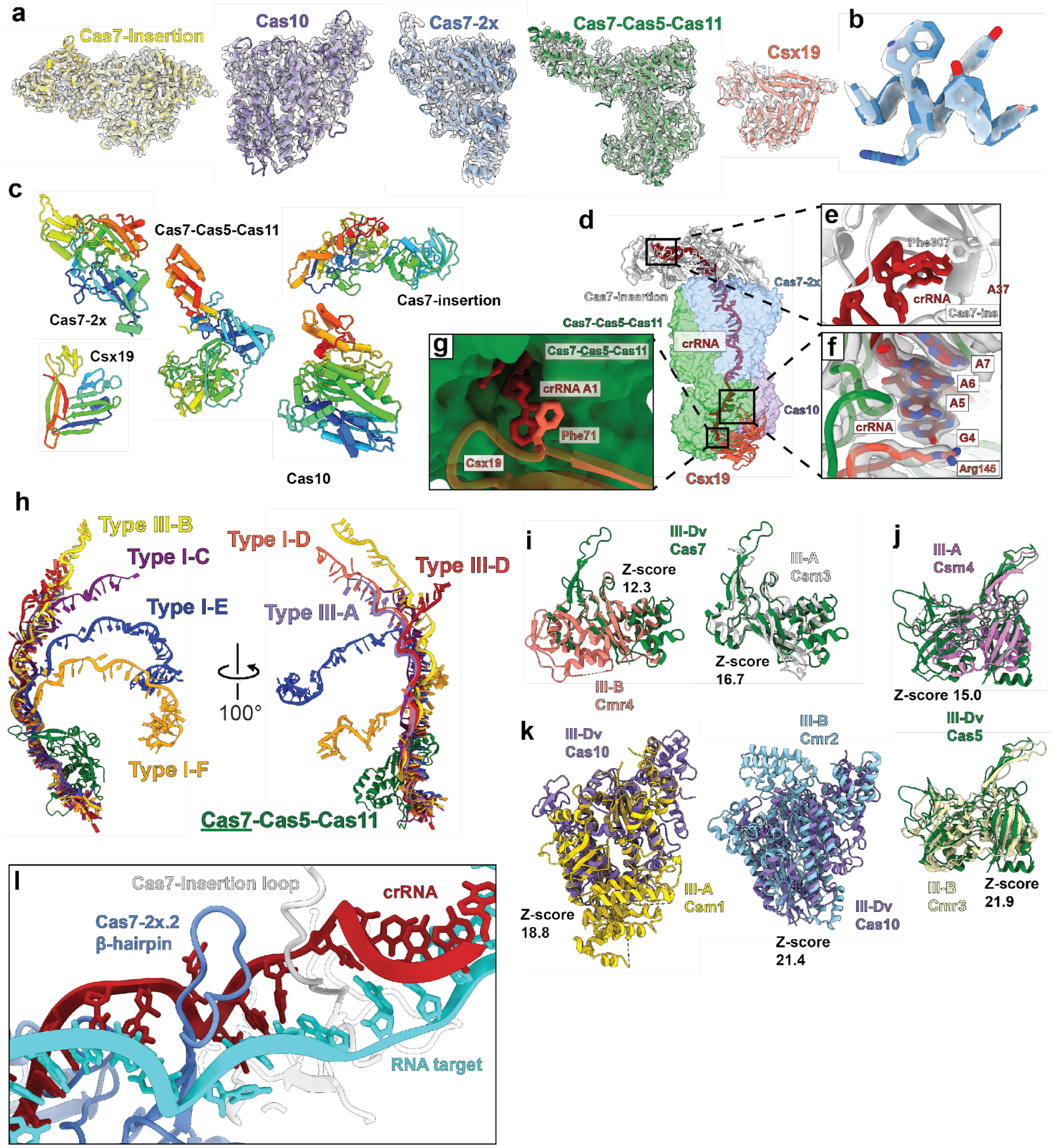

Supplementary Fig. 5 | Analysis of type III-Dv subunits, crRNA, and RNA target. a, Individual subunits fit into their respective cryo-EM density. **b,** Density of an alpha-helix in the Cas7-insertion subunit. **c,** Connectivity of the type III-Dv subunit structures. The subunits are colored N-terminus (blue) to C-terminus (red). **d,** crRNA trajectory through the type III-Dv effector complex. **e,** 3' crRNA capping by Phe307 of Cas7-insertion. **f,** 5' crRNA capping by Phe71 of Csx19. **g,** Arg145 of Csx19 interacting with G4 of the crRNA, upstream of the 5' crRNA handle. **h,** crRNA geometry comparison between type III-Dv and other type I and type III systems. **i,** Structural alignments between the III-Dv Cas7 domain of Cas7-Cas5-Cas11 with Cmr4 (Cas7) of the type III-B (peach, Z-score 12.3) and Csm3 of the type III-A complex (white, Z-score 16.7); **j,** III-Dv Cas5 domain of

Cas7-Cas5-Cas11 with Cas5 (Csm4) of the type III-A complex (magenta, Z-score 15.0) and Cas5 (Cmr3) of the type III-B complex (beige, Z-score 21.9); **k,** III-Dv Cas10 subunit with Cas10 (Csm1) of the type III-A complex (yellow) and Cas10 (Cmr2) of the type III-B complex (cyan). **l,** Comparison between the protrusion of the Cas7-2x.2 beta-hairpin and Cas7-insertion loop between the crRNA and the RNA target duplex.

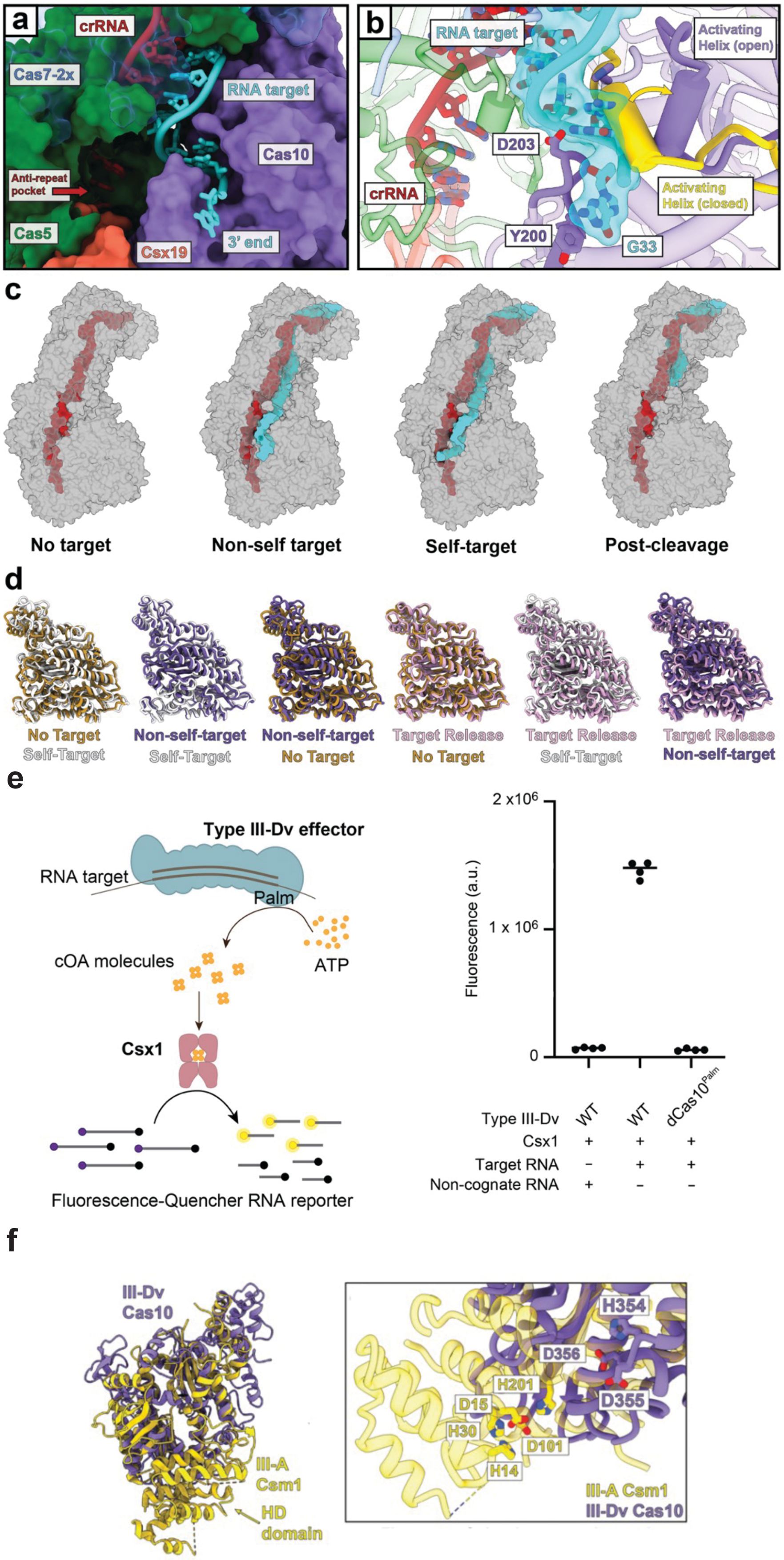
a
crRNA
Cas7-2x
RNA target
Cas10
Anti-repeat pocket
Cas5
Csx19
3' end
b
RNA target
Activating Helix (open)
D203
crRNA
Activating Helix (closed)
Y200
G33
c
No target
Non-self target
Self-target
Post-cleavage
d
No Target
Self-Target
Non-self-target
Self-Target
Non-self-target
No Target
Target Release
No Target
Target Release
Self-Target
Target Release
Non-self-target
e
Type III-Dv effector
RNA target
Palm
ATP
cOA molecules
Csx1
Fluorescence-Quencher RNA reporter
2 x10^6
1 x 10^6
0
Fluorescence (a.u.)
Type III-Dv
WT
WT
dCas10^Palm
Csx1
+
+
+
Target RNA
–
+
+
Non-cognate RNA
+
–
–
f
III-Dv Cas10
III-A Csm1
HD domain
H354
D356
H201
D15
D355
H30
D101
H14
III-A Csm1
III-Dv Cas10

Supplementary Fig. 6 | Cas10 activation by non-self RNA binding. a, Path of the RNA target strand gets directed through Cas10 rather than into the anti-repeat pocket. **b,** Upon binding a non-self RNA target, an activating helix in Cas10 gets pushed by the target RNA into an active conformation. Yellow arrow indicates directionality of movement of this alpha helix. **c,** Structures of the type III-Dv complex before target binding, after non-self- and self-target binding, and in a post-cleavage state. **d,** Conformational changes within Cas10 between the different structures described in **c. e,** Specific binding by the type III-Dv complex activates Csx1 accessory RNase via its Palm domain. *Left,* Schematic representation of RNA reporter-based assay to demonstrate type III-Dv/Csx1 coupled fluorescence. *Right,* Fluorescence intensities of type III-Dv/Csx1 coupled assay. Either wild-type type III-Dv complex (WT) or modified complex with mutated Palm domain (dCas10^{Palm}; Cas10 modifications D308A, D309A) incubated with target or non-cognate RNA. Fluorescence was measured after 5 min reaction time. Mean of 4 replicates are shown. **f,** *Left,* Comparison of the type III-Dv Cas10 subunit (purple) with type III-A Cas10 (Csm1) (yellow; PDB 6o74). *Right,* HD domain comparison.

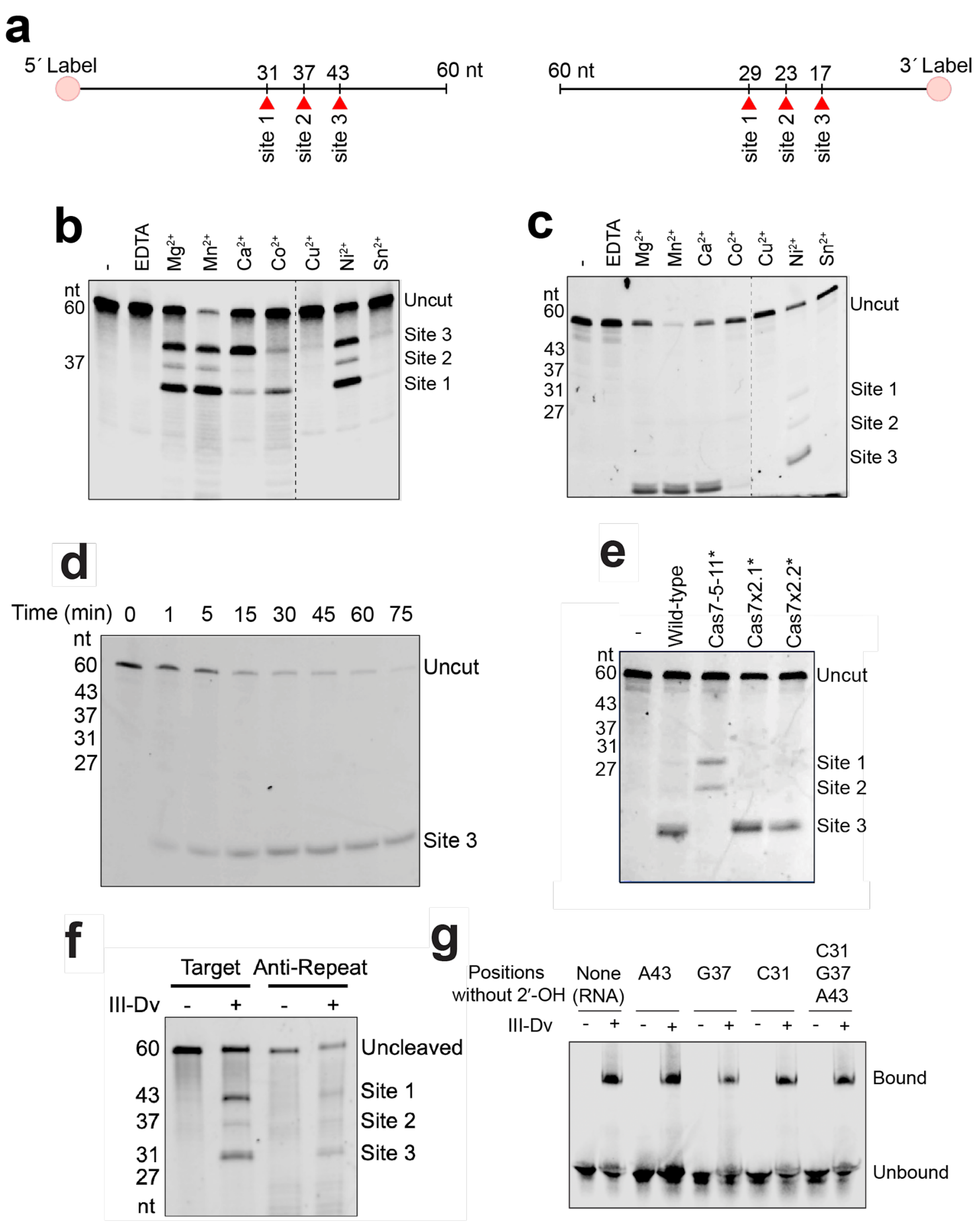

Supplementary Fig. 7 | Electrophoretic Mobility Shift Assays of RNA target cleavage and binding type III-Dv. a, Fluorescent probes used in RNA cleavage reactions. **b,** Metal dependent cleavage of the 5'-labelled RNA target. Cleavage site 1, 2, and 3 correspond to 31, 37, and 43 nucleotide products, respectively. **c,** Metal dependent cleavage of the 3'-labeled RNA target. Cleavage site 1, 2, and 3 correspond to 29, 23, and 17 nucleotide products, respectively. **d,** RNA cleavage time course with a 3'-labeled RNA target across 75 minutes. **e,** 3'-labeled RNA cleavage analysis after mutagenesis of the three active site residues of Cas7-Cas5-Cas11, Cas7-2x.1, and Cas7-2x.2. **f,** Comparison of RNA cleavage of Non-Self RNA target (left) and Self RNA target (right). **g,** Binding of a DNA-RNA hybrid target by the type III-Dv complex with 2'-OH groups removed at specified positions.

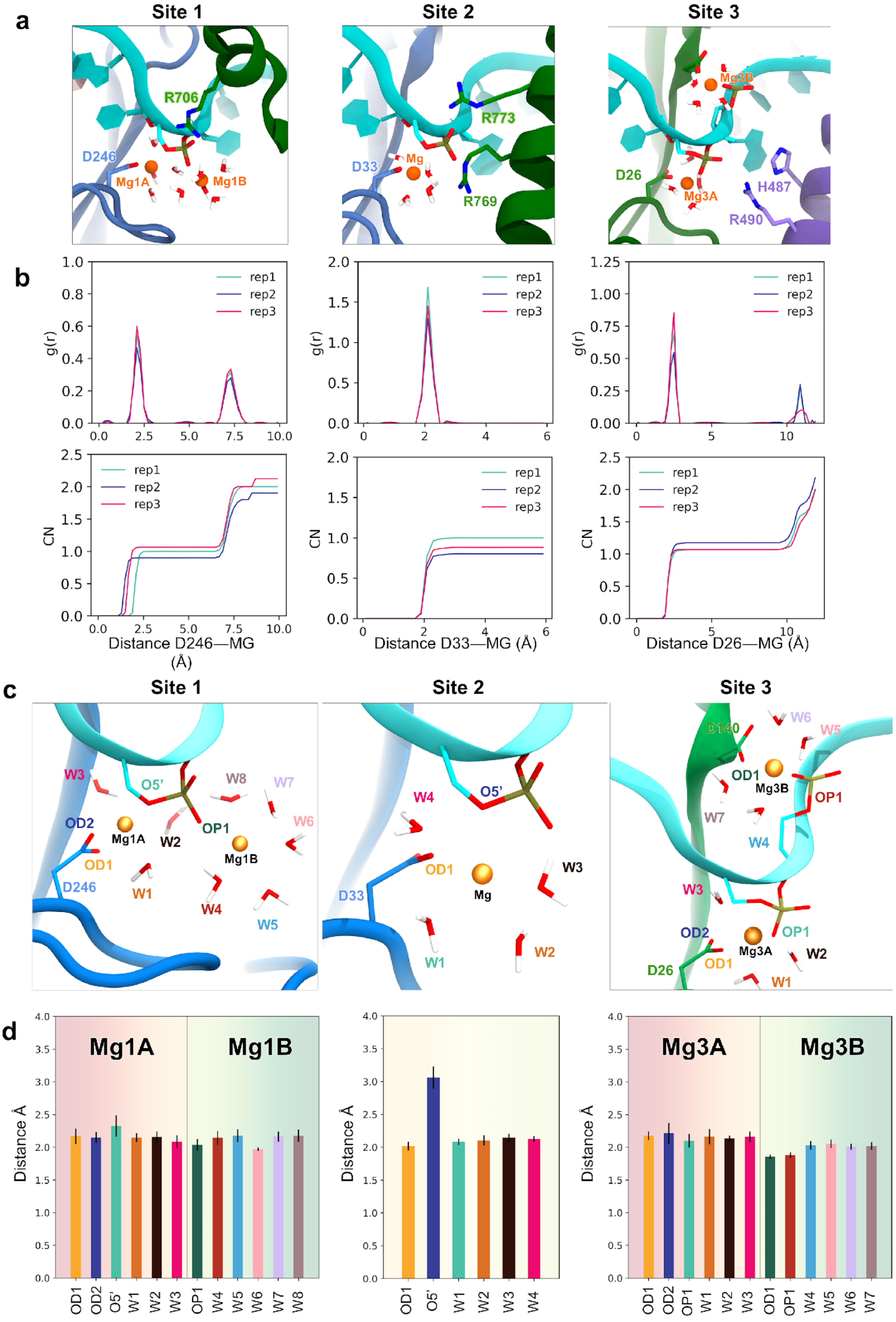
a
Site 1
Site 2
Site 3
R706
D246
Mg1A
Mg1B
R773
D33
Mg
R769
Mg3B
D26
Mg3A
H487
R490
b
g(r)
rep1
rep2
rep3
CN
Distance D246—MG (Å)
Distance D33—MG (Å)
Distance D26—MG (Å)
c
Site 1
Site 2
Site 3
W3
O5'
W8
W7
W6
OD2
Mg1A
W2
OP1
Mg1B
OD1
D246
W1
W4
W5
W4
O5'
OD1
Mg
W3
D33
W1
W2
W6
W5
D140
OD1
Mg3B
OP1
W7
W4
W3
OD2
Mg3A
OP1
W2
D26
OD1
W1
d
Mg1A
Mg1B
Mg3A
Mg3B
Distance Å
OD1
OD2
O5'
W1
W2
W3
OP1
W4
W5
W6
W7
W8

Supplementary Fig. 8 | Analysis of the three active sites of the type III-Dv complex. a, Representative snapshot showing the placement of Mg^{2+} ion at each active site of type III-Dv CRISPR-Cas effector complex during μs-long MD simulation. **b,** Radial distribution function g(r) and coordination number calculated from the all-atom MD simulation shows sites 1 and 3 can accommodate two Mg^{2+} ions while site 2 takes in only one Mg^{2+} ion. These results were consistent in all three replicas. **c,** The coordination of the metal ions attained during ~40 ps of ab-initio QM(Car-Parrinello)/MM MD is shown for the three active sites. **d,** Distances between the Mg^{2+} ion and key atoms involved in its coordination is shown for each catalytic site, distance is the average of stable last ~20 ps of QM/MM simulation and error bars represent standard deviation.

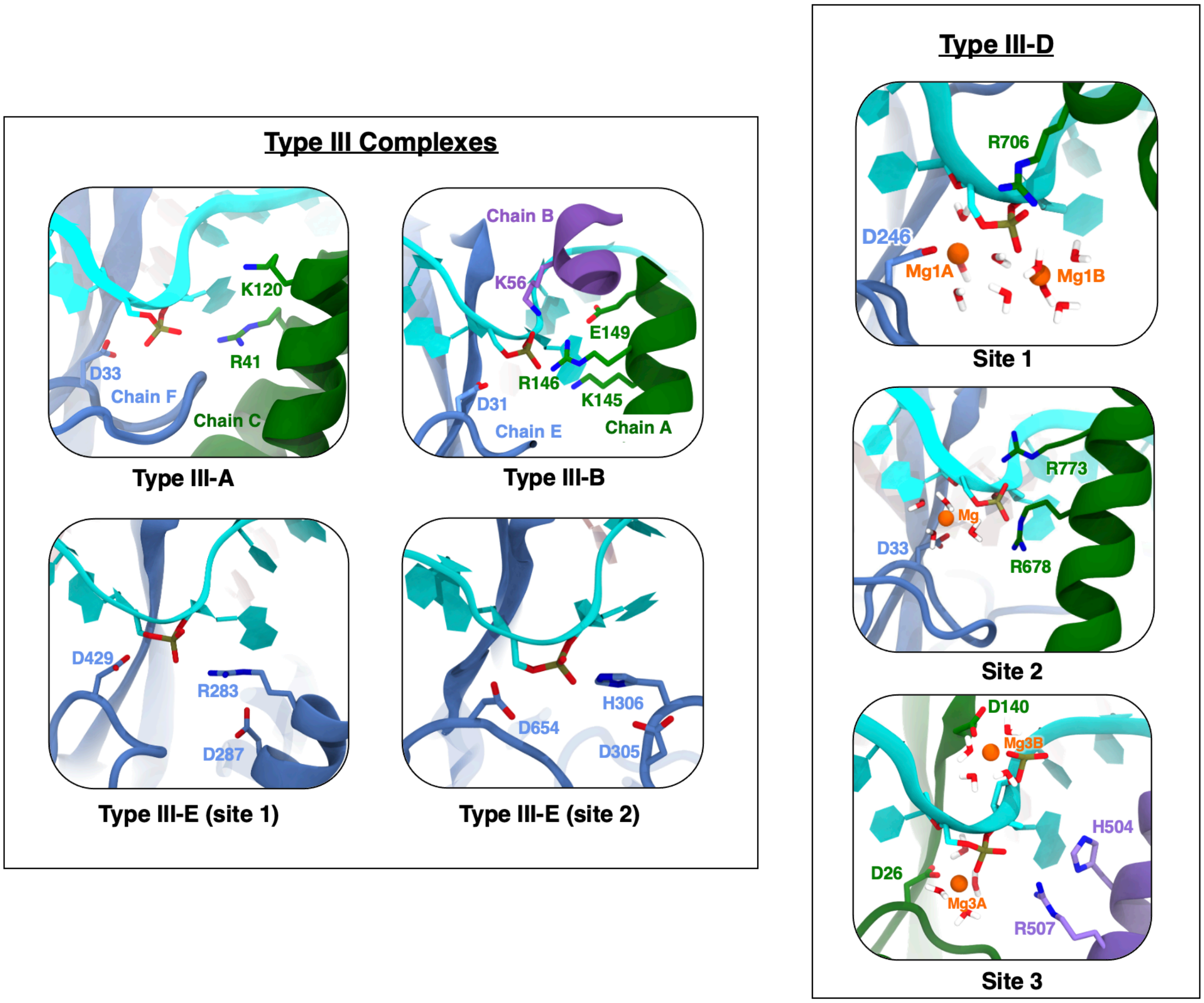

Supplementary Fig. 9 | Active site comparisons between the type III-Dv complex and other type III complexes. *Left*, Cas7 active sites from type III-A, -B and -E complexes. *Right,* Cas7 active sites from type III-Dv complex. The structural arrangement of active site 2 from the type III-Dv complex is similar to active sites from other type III complexes.

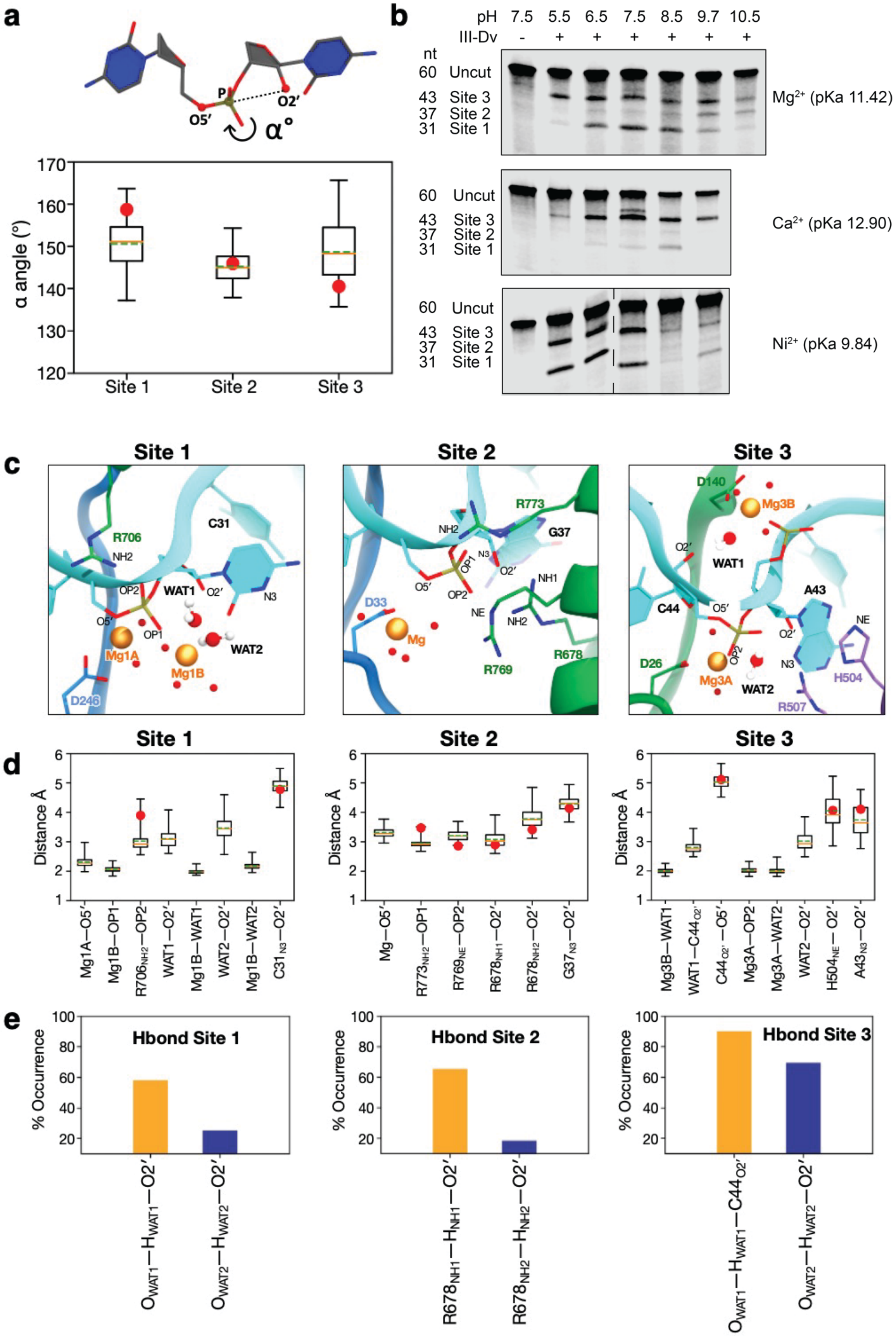
a
P
O5′
O2′
α°
α angle (°)
Site 1
Site 2
Site 3
b
pH 7.5 5.5 6.5 7.5 8.5 9.7 10.5
III-Dv - + + + + + +
nt
60 Uncut
43 Site 3
37 Site 2
31 Site 1
Mg2+ (pKa 11.42)
Ca2+ (pKa 12.90)
Ni2+ (pKa 9.84)
c
Site 1
R706
NH2
C31
OP2
WAT1
O2′
N3
O5′
OP1
WAT2
Mg1A
Mg1B
D246
Site 2
R773
NH2
G37
N3
O2′
OP1
O5′
OP2
NH1
NE
NH2
D33
Mg
R769
R678
Site 3
D140
Mg3B
O2′
WAT1
C44
O5′
A43
NE
O2′
N3
D26
OP2
Mg3A
WAT2
H504
R507
d
Site 1
Distance Å
Mg1A—O5′
Mg1B—OP1
R706NH2—OP2
WAT1—O2′
Mg1B—WAT1
WAT2—O2′
Mg1B—WAT2
C31N3—O2′
Site 2
Mg—O5′
R773NH2—OP1
R769NE—OP2
R678NH1—O2′
R678NH2—O2′
G37N3—O2′
Site 3
Mg3B—WAT1
WAT1—C44O2′
C44O2′—O5′
Mg3A—OP2
Mg3A—WAT2
WAT2—O2′
H504NE—O2′
A43N3—O2′
e
% Occurrence
Hbond Site 1
OWAT1—HWAT1—O2′
OWAT2—HWAT2—O2′
Hbond Site 2
R678NH1—HNH1—O2′
R678NH2—HNH2—O2′
Hbond Site 3
OWAT1—HWAT1—C44O2′
OWAT2—HWAT2—O2′

Supplementary Fig. 10 | Structural and biochemical analysis supporting catalysis. a. Model for the inline nucleophilic attack by the 2'OH nucleophile to the scissile phosphate P. To ensure effective overlap between the lone pairs of the O2' and the antibonding orbital of the P−O5' bond, the α angle between O2'−P−O5' should lie within the 140°-180° range. The box plot reports the value of the α angle at each active site, computed from the QM/MM trajectories. The green dashed line reports the mean values, and the red dot represents the value in the cryo-EM structure. **b.** 5'IRD800-labeled RNA cleavage analysis at varying pH levels of the three active sites examining the activity for similarly-sized divalent metal ions that have diverse pKa values. **c.** Close-up view of the three active sites of the type III-Dv complex, as observed during QM/MM simulations. The first-shell water molecules, hydrogen bonding to the O2' nucleophile, are shown as sticks & balls. **d.** Boxplots showing the distribution of critical distances within the three active sites, as from computed from the QM/MM trajectories. The green dashed line reports the mean values, and the red dot represents the value in the cryo-EM structure. **e.** Occurrence of hydrogen bonds (expressed as % of their number over the total number of frames) participating in γ' catalysis for three active sites, as from QM/MM simulations. Hydrogen bonds are defined using a distance cutoff of 3.5 Å and an angle greater than 140°.
